# Model-Free Adaptive Sensing and Control for a Piezoelectrically Actuated System

**DOI:** 10.3390/s101210545

**Published:** 2010-11-24

**Authors:** Hung-Yi Chen, Jin-Wei Liang

**Affiliations:** Ming Chi University of Technology/No. 84, Gungjuan Road, Taishan, Taipei, 243, Taiwan; E-Mail: liangj@mail.mcut.edu.tw

**Keywords:** piezoelectrically actuated system, functional approximation technique, adaptive sliding controller

## Abstract

Since the piezoelectrically actuated system has nonlinear and time-varying behavior, it is difficult to establish an accurate dynamic model for a model-based sensing and control design. Here, a model-free adaptive sliding controller is proposed to improve the small travel and hysteresis defects of piezoelectrically actuated systems. This sensing and control strategy employs the functional approximation technique (FAT) to establish the unknown function for eliminating the model-based requirement of the sliding-mode control. The piezoelectrically actuated system’s nonlinear functions can be approximated by using the combination of a finite number of weighted Fourier series basis functions. The unknown weighted vector can be estimated by an updating rule. The important advantage of this approach is to achieve the sliding-mode controller design without the system dynamic model requirement. The update laws for the coefficients of the Fourier series functions are derived from a Lyapunov function to guarantee the control system stability. This proposed controller is implemented on a piezoelectrically actuated X-Y table. The dynamic experimental result of this proposed FAT controller is compared with that of a traditional model-based sliding-mode controller to show the performance improvement for the motion tracking performance.

## 1. Introduction

Recently, the micro-positioner has become an important development target for meeting the requirements of the precision industry, such as in the semiconductor manufacturing process, biotechnology processes and opto-electronics systems. Since the piezoelectric actuator has many advantages, such as ultrahigh precision, high resolution, tiny size and quick response speed, it has been widely used as a micro-positioning table actuator in these production areas. Piezoelectric actuators can also be electrically controlled to move with a resolution on the order of nanometers. However, piezoelectric actuators also exhibit undesired serious hysteretic behaviors which limit the performance of piezoelectric actuated systems.

In recent years, different methods were proposed to drive the piezoelectric micropositioning mechanisms and control piezoelectrically actuated systems. Chang and Sun [1] tried to control a two degree-of-freedom monolithic piezoelectric actuator with a linear resolution to 2 nm. Choi *et al*. [2] designed a sliding-mode controller for fine motion tracking control of the objective lens in the vertical direction. Lin and Yang [3] employed a PI feedback control associated with feedforward compensation based on the hysteresis observer to compensate the nonlinearity of piezoelectric actuator. Bashash and Jalili [4] proposed a modeling and control methodology for real-time compensation of nonlinearities along with precision trajectory control of piezoelectric actuators in various range of frequency operation. Liaw *et al*. [5] presented a robust motion tracking control methodology for flexure-based four-bar micro/nano manipulator driven by a piezoelectric actuator. In addition, many control techniques involving feedback and feedforward-feedback features have been proposed to remove the hysteresis-caused tracking errors [6–10]. However, these feedback control techniques do not utilize a precise hysteresis model, in contrast, the feedforward-feedback algorithms deal with the hysteresis and structural dynamics separately. A compensator is added into the feedforward loop to cancel out the undesired effects caused by the hysteresis while designing a feedback controller to control the structural dynamic effects. The feedforward compensator can be used to deal with nonlinear hysteretic effects; however, the effectiveness of the method relies heavily on the accuracy of the inverse hysteresis model.

Although a piezoelectric actuator has the advantages of high resolution, quick response speed and high power-volume ratio, it has serious hysteresis behavior and small travel defects. Hence, it is difficult to establish an accurate dynamic model for model-based control design. In this paper, a model-free functional approximation based adaptive sliding-mode controller is employed to eliminate this model-based requirement. The functional approximation technique (FAT) was utilized to design an adaptive sliding controller for different nonlinear systems containing time-varying uncertainties [11–13]. This control scheme is proposed for nonlinear systems with unknown bound time-varying uncertainties. This approach can estimate the system dynamics and uncertainties on-line and no prior knowledge of the system dynamics is required. The uncertainties are represented by using finite linear combinations of basis functions with unknown constant weighting vectors. Appropriate update laws for the weighting vectors can be selected based on the Lyapunov theory to guarantee the system stability. Asymptotic convergence of the output error can be obtained if sufficient number of basis functions is used. The optimal expansion coefficients are also updated on-line and used in constructing the adaptive control input. Here, the functional approximation based adaptive sliding-mode controller is designed for a piezoelectric actuated X–Y table system tracking control.

This paper is organized as follows: Section 2 describes the piezoelectrically actuated X–Y table system structure. The approximate linear model and system identification process are presented in Section 3. This identified model can be used to design the model-based controller for comparison. Section 4 describes the functional approximation technique. The methodology of controller design and the stability analysis are derived in Section 5. Section 6 describes the experimental results of the proposed FAT controller. The experimental results are compared with that of the model-based sliding controller to show the dynamic performance improvement of the proposed FAT controller. Final conclusions are presented in Section 7.

## 2. The Experimental System Structure

**Figure 1.**
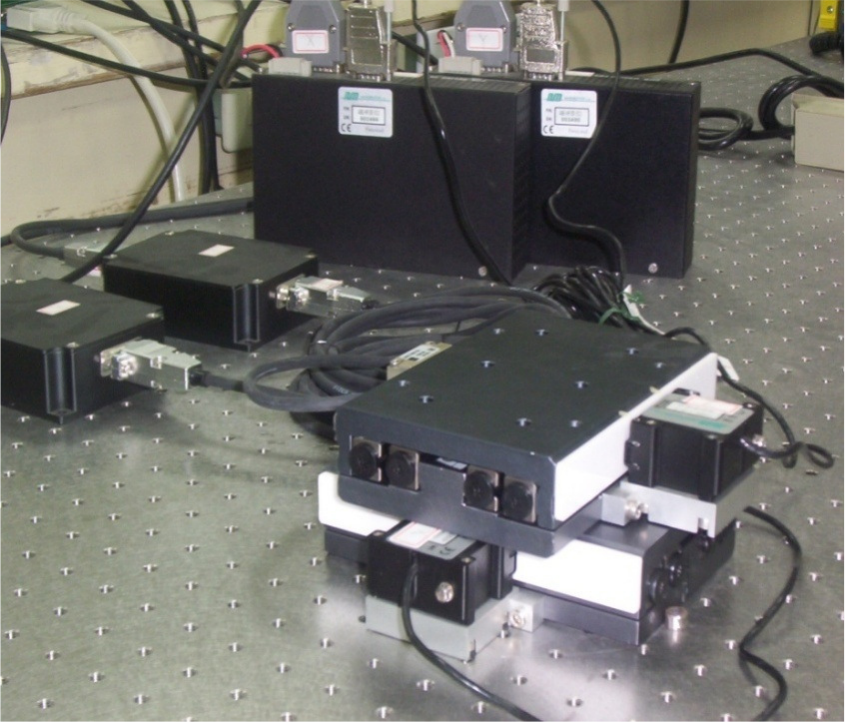
Piezoelectrically actuated X–Y table system.

A piezoelectrically actuated X–Y table shown in Figure 1 was built for investigating the dynamic system control behavior. Two HR8 motors were used to actuate the X axis and Y axis, respectively. There is a linear response feature between the piezoelectric actuator velocity and the driver control voltage. The actuator and driver can be modeled as a DC motor with internal friction that is driven by a voltage amplifier. The driver generates a 39.6 kHz sinusoidal wave to drive the actuator with an amplitude function when a command voltage within ±10 V is sent to the driver unit. This constant oscillation frequency is generated from the driver unit which was supplied by Nanomotion Limited.

**Figure 2.**
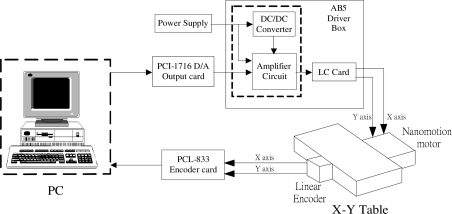
Positioning control system experimental layout.

A PC-based controller was developed for experiments with this system. The X–Y table has two independent axes, X and Y, actuated by two different piezoelectric actuators. The experimental layout of this positioning system is shown in Figure 2. The control voltage is calculated on the PC, converted from a digital to an analogue signal by a D/A interface card and sent to the piezoelectric actuator driver unit to actuate the piezoelectric motor. The displacements of this X–Y table are sensing and measured by linear encoders and sent back to the PC via an encoder card for closed-loop control.

To simplify the model description, the system dynamics of the X axis or Y axis can be represented as the following second order model:

(1) $$\ddot{x}(t)=f(X,\;t)+b(t)u(t)$$

where *x* is the displacement of X axis or Y axis stage, *f*(*X*,*t*) is a function of state variables, *b(t)* is the control gain and *u(t)* is the control voltage. The function *f*(*X*,*t*) is an unknown time-varying function with unknown variation bound. However, the bound of the unknown function *b(t)* can be estimated, *i.e.*, *b*_min_≤*b(t)*≤*b*_max_ Define *b(t)* as:

(2) $$b(t)=b_{m}+\Delta b$$

where *b_m_* is the nominal value and Δ*b* is a bounded uncertainty value:

(3) $$0<\tau_{\text{min}}\leq \Delta b\leq \tau_{\text{max}}$$

Since the system dynamics has nonlinear time-varying behavior with unknown uncertainty bounds it is difficult to establish an accurate dynamic model for model-based controller design. Here, the functional approximation technique is employed to approximate this unknown function *f*(*X*,*t*) for releasing the model requirement.

## 3. System Identification

In order to evaluate the accuracy of a functional approximation with respect to the nonlinear function of the system, an approximate linear dynamic model for this piezoelectrically actuated X–Y table, instead of the original system model, is identified based on system input-output data. A pseudo-random-binary-sequences (PRBS) signal with appropriate amplitude is chosen as the input signal to excite the piezoelectrically actuated system. The transfer function *M*(*q*) describes the relationship between the voltage input and the sprung mass position output is chosen as an auto-regressive (ARX) model:

(4) $$M(q)=\frac{B(q)}{A(q)}$$

where *A*(*q*) = 1 + *a*_1_*q*^−1^ *+* … + *a_n_a__* *q*^−*n*_*z*_^, *B*(*q*) *= b*_1_*q*^−1^ + … + *b_n_b__* *q*^−*n*_*b*_^ and *q* is the shift operator. To simplify the model description, the second order approximate transfer function models are selected and identified by using the MATLAB’s identification toolbox for X axis and Y axis of the X–Y table, respectively:

(5) $$M_{x}(q)=\frac{78.24}{q^{2}+0.7202q+0.3588}$$

(6) $$M_{y}(q)=\frac{16.8}{q^{2}+0.2815q+0.4079}$$

**Figure 3.**
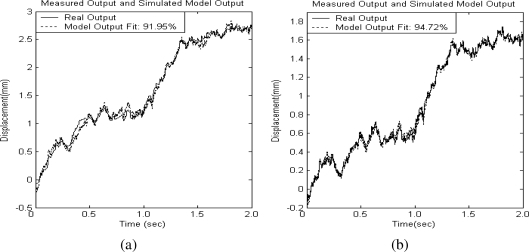
System identification model output. **(a)** X axis; **(b)** Y axis.

The estimation output of these two identified models and the system experimental output with PRBS input voltage excitations are plotted in Figures 3(a) and 3(b) for comparison. The solid line represents the measured system output and the dotted line depicts the simulated model response. It can be observed that the dynamic behavior of the identified model is matched well the piezoelectrically actuated system’s dynamic response. The difference between both response curves is due to the system’s nonlinear, time-varying behavior and hysterisis dynamics effects. Based on these identified models, the unknown time-varying functions can be estimated and used to evaluate the accuracy of the functional approximation scheme. In addition, a model-based sliding-mode controller can be designed based on the estimation models. Its control performance will be compared with that of the proposed FAT controller.

## 4. Functional Approximation Technique

If a piecewise continuous time varying function *h*(*t*) satisfies the Dirichlet conditions, it can be transformed into a generalized Fourier series expansion within a time interval [0,*T*]:

(7) $$h(t)=a_{0}+\sum_{n=1}^{\infty }\left(a_{n}\text{cos}\omega_{n}t+b_{n}\text{sin}\omega_{n}t\right)$$

where *a*_0_, *a_n_*, and *b_n_* are the Fourier coefficients and $\omega_{n}=\frac{2n\pi }{T}$ is the frequency of the sinusoidal function. Define:

(8) $$Z(t)={\left[1\;\;\;\;\;\text{cos}\omega_{1}t\;\;\;\;\;\text{sin}\omega_{1}t\;\;\;\;\;\text{cos}\omega_{2}t\;\;\;\;\;\text{sin}\omega_{2}t\;\;\;\cdots \;\;\;\text{cos}\omega_{n}t\;\;\;\;\;\text{sin}\omega_{n}t\right]}^{T}$$

(9) $$W={\left[a_{0}\;\;\;\;a_{1}\;\;\;\;b_{1}\;\;\;\;a_{2}\;\;\;\;b_{2}\;\;\;\;\cdots \;\;\;\;a_{n}\;\;\;\;b_{n}\right]}^{T}$$

then Equation (7) can be rewritten as:

(10) $$h(t)=W^{T}Z(t)+ɛ(t)$$

where ɛ(*t*) is the approximation error. When *n* is large enough, *h*(*t*) can be approximated as:

(11) $$h(t)\approx W^{T}Z(t)$$

The unknown time-varying function *f*(*X*,*t*) in Equation (1) can be approximated by a linear combination of a finite orthogonal basis functions *Z*(*t*) to arbitrarily prescribed accuracy as long as *n* is large enough:

(12) $$f(X,\;t)\approx W_{f}^{T}Z_{f}(t)$$

where *Z_f_*(*t*) is a orthogonal basis function vector and *W_f_* is a weighting coefficient vector. If the number of the basis functions is large enough, Equation (12) can be described as the following approximation form:

(13) $$f(X,\;t)=W_{f}^{T}Z_{f}(t)$$

where *Z_f_* (*t*) = [*Z*_1_(*t*) *Z*_2_(*t*) … *Z_n_*(*t*)]*^T^*, and *W_f_* = [*W*_1_ *W*_2_ … *W_n_*]*^T^*. This functional approximation in Equation (13) can be used to represent an unknown function with uncertainty and disturbance. The time-varying vector *Z_f_*(*t*) is a known function and the vector *W_f_* is an unknown regulating constant. A proper Lyapunov function can be selected to find the update laws for these unknown constants based on Lyapunov stability theory.

## 5. Controller Design and Stability Analysis

The objective of this study was to develop a FAT based model-free adaptive sliding-mode controller for a piezoelectrically actuated system. Its control performance is compared with that of a traditional sliding-mode controller based on an identified model, Equations (5) and (6). The control law design and stability analysis for X axis are described in the following sections.

### 5.1. Functional Approximation Based Adaptive Sliding-Mode Controller

**Figure 4.**
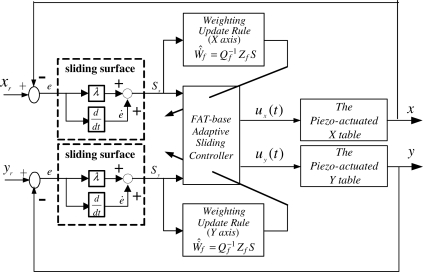
System control block diagram.

Due to the piezoelectrically actuated system has nonlinear time-varying dynamics, the functional approximation technique is employed to replace the unknown nonlinear functions for a sliding-mode control design. The system control block diagram of this piezoelectrically actuated X–Y table is shown in Figure 4. The adaptive laws of the function coefficients can be derived from Lyapunov stability theorem. The sliding surface of this second order system can be defined as:

(14) $$s=\dot{e}+\lambda e$$

where the positive parameter *λ* implies the convergent rate of *x* on the sliding surface. The time derivative of *s* can be derived as:

(15) $$\begin{array}{l} \dot{s}=\ddot{e}+\lambda \dot{e}\\ \;\;\;=\ddot{x}-{\ddot{x}}_{r}+\lambda \dot{e} \end{array}$$

where *x_r_* is the desired value of *x*. Substituting Equation (1) into Equation (15), yields:

(16) $$\dot{s}=f(x,\;t)+b(t)u-{\ddot{x}}_{r}+\lambda \dot{e}$$

In order to achieve the sliding surface reaching condition and establish the approximation error compensation, the control law *u(t)* can be designed as:

(17) $$u=\frac{1}{b_{m}}(-\hat{f}+{\ddot{x}}_{r}-\lambda \dot{e}-\eta \text{sgn}(s))$$

where *f̂* is the functional approximation value of *f*(*X*,*t*) The positive constant *η* is a design parameter for achieving an appropriate robustness. Substituting Equation (17) into Equation (16), we obtain:

(18) $$\dot{s}=-\eta \text{sgn}(s)+(f-\hat{f})+\Delta bu$$

Here, *f* and *f̂* are assumed to be unknown bounded piece-wise continuous functions and satisfy the Dirichlet conditions. Then, they can be presented by the functional approximation technique as:

(19) $$f=W_{f}^{T}Z_{f}$$

(20) $$\hat{f}={\hat{W}}_{f}^{T}Z_{f}$$

where *W_f_*,*Ŵ_f_* ∈ ℜ*^n^* are weighting vectors and *Z_f_* ∈ ℜ*^n^* is a vector of basis Fourier series function. Hence, the Equation (18) can be rewritten as:

(21) $$\dot{s}=-\eta \text{sgn}(s)+{\tilde{W}}_{f}^{T}Z_{f}+\Delta bu$$

where:

(22) $${\tilde{W}}_{f}^{T}=W_{f}^{T}-{\hat{W}}_{f}^{T}$$

To prove the stability of this control system and find the update laws for vector *Ŵ_f_*, a Lyapunov function candidate is chosen as:

(23) $$V(s,\;{\tilde{W}}_{f})=\frac{1}{2}s^{2}+\frac{1}{2}{\tilde{W}}_{f}^{T}Q_{f}{\tilde{W}}_{f}$$

where *Q_f_* ∈ ℜ*^n×n^* is a symmetric positive definite matrix. By taking the time derivative of the Lyapunov function candidate, we can obtain:

(24) $$\dot{V}(s,\;{\tilde{W}}_{f})=s\dot{s}+{\tilde{W}}_{f}^{T}Q_{f}{\dot{\tilde{W}}}_{f}$$

Since ${\dot{\tilde{W}}}_{f}^{T}=-{\dot{\hat{W}}}_{f}^{T}$, the Equation (24) can be rewritten as:

(25) $$\begin{array}{l} \dot{V}(s,\;{\tilde{W}}_{f})=s(-\eta \text{sgn}(s)+{\tilde{W}}_{f}^{T}Z_{f}+\Delta bu)+{\tilde{W}}_{f}^{T}Q_{f}{\dot{\tilde{W}}}_{f}\\ \;\;\;\;\;\;\;\;\;\;\;\;\;\;\;\;\;\;\;\;\;\;\;\;\;\;\;\;=-\eta |s|+{\tilde{W}}_{f}^{T}(Z_{f}s-Q_{f}{\dot{\hat{W}}}_{f})+\Delta bus \end{array}$$

The update law for *Ŵ_f_* is chosen as:

(26) $${\dot{\hat{W}}}_{f}=Q_{f}^{-1}Z_{f}s$$

Then, the Equation (25) can be further rewritten as:

(27) $$\dot{V}=-\eta |s|+\Delta bus$$

In order to cover the uncertainty of the unknown function *b*(*t*), and establish an appropriate robustness, the parameter η can be specified as:

(28) $$\eta =\tau_{\text{max}}u_{\text{max}}$$

where *τ*_max_ and *u*_max_ are the maximum values of Δ*b* and *u*, respectively. Then the Equation (27) results in:

(29) $$\dot{V}\leq 0$$

That means this control system stability can be guaranteed by using the update laws shown in Equation (26). Based on Barbarlet’s lemma [14], the convergence of the system output error can be guaranteed by using the control law *u*(*t*), Equation (17). The design procedure of the controller for Y axis is similar to that of X axis.

### 5.2. Model-Based Sliding-Mode Controller Design

The sliding-mode control theory has been widely employed to control nonlinear dynamic systems, especially the systems that have model uncertainties and external disturbances. It employs a discontinuous control effort to drive the system toward a sliding surface, and then switching on that surface. Theoretically, it will gradually approach the control target, the origin of the phase plane [15,16]. In this section, a model-based sliding-mode controller is designed for the X axis of the piezoelectrically actuated system based on the estimated models. Firstly, the time-varying second order system model is approximately represented as the form:

(30) $$\begin{array}{l} \ddot{x}=-A\dot{x}(t)-Bx(t)+Gu(t)+D(t)\\ \;\;\;\;=-(A_{m}+\Delta A)\dot{x}-(B_{m}+\Delta B)x+(G_{m}\cdot \Delta G)u+(D_{m}+\Delta D) \end{array}$$

These system parameters have time-varying behavior. Their variation bounds are assumed as:

(31) $$\{\begin{array}{c} |\Delta A|<\alpha \\ |\Delta B|<\beta \\ |\Delta D|<\gamma \\ 0\leq \delta_{\text{min}}=\frac{G_{\text{min}}}{G_{m}}\leq \Delta G\leq \frac{G_{\text{max}}}{G_{m}}=\delta_{\text{max}} \end{array}$$

The sliding surface for this second order system can be defined as:

(32) $$s_{s}=\dot{e}+\lambda_{s}e$$

where the positive constant *λ_s_* implies the convergent rate of *x* on the sliding surface. Substituting Equation (30) into the time derivative of *s_s_*, we obtain:

(33) $${\dot{s}}_{s}=-(A_{m}+\Delta A)\dot{x}-(B_{m}+\Delta B)x+(G_{m}\cdot \Delta G)u_{s}+(D_{m}+\Delta D)-{\ddot{x}}_{r}+\lambda_{s}\dot{e}$$

The control law *u_s_*(*t*) can be chosen as:

(34) $$u_{s}=\frac{1}{G_{m}}[A_{m}\dot{x}+B_{m}x-D_{m}+{\ddot{x}}_{r}-\lambda_{s}\dot{e}-\eta_{1}\text{sgn}(s_{s})]$$

Substituting Equation (34) into Equation (33), gives:

(35) $$\begin{array}{ll} {\dot{s}}_{s} & =(1-\Delta G)[-A_{m}\dot{x}-B_{m}x+D_{m}-{\ddot{x}}_{r}+\lambda_{s}\dot{e}]-\Delta A\dot{x}-\Delta Bx+\Delta D-\Delta D\eta_{1}\text{sgn}(s_{s})\\ & \;=(1-\Delta G)\hat{u}-\Delta A\dot{x}-\Delta Bx+\Delta D-\Delta G\eta_{1}\text{sgn}(s_{s}) \end{array}$$

where:

(36) $${\hat{u}}_{s}=-A_{m}\dot{x}-B_{m}x+D_{m}-{\ddot{x}}_{r}+\lambda_{s}\dot{e}$$

Multiplying both sides of Equation (35) with the sliding variable *s_s_*, it can be written as:

(37) $$\begin{array}{l} s_{s}{\dot{s}}_{s}=(1-\Delta G){\hat{u}}_{s}s_{s}-{\Delta A\dot{x}s}_{s}-{\Delta Bxs}_{s}+{\Delta Ds}_{s}-{\Delta G\eta }_{1}\text{sgn}|s_{s}|\\ \;\;\;\;\;\;\;\;\leq (1-\delta_{\text{min}})|{\hat{u}}_{s}||s_{s}|+\alpha |\dot{x}||s_{s}|+\beta |x||s_{s}|+\gamma |s_{s}|-\delta_{\text{min}}\eta_{1}|s_{s}| \end{array}$$

If the robustness parameter η_1_ is selected as:

(38) $$\eta_{1}\geq \frac{1}{\delta_{\text{min}}}[(1-\delta_{\text{min}})|{\hat{u}}_{s}|+\alpha |\dot{x}|+\beta |x|+\gamma +\eta_{2}],\;\;\eta_{2}>0$$

Then the Equation (37) results in:

(39) $$s_{s}{\dot{s}}_{s}\leq -\eta_{2}|s_{s}|<0$$

That means the system stability can be achieved by choosing an appropriate robustness gain constant η_1_. In addition, the control law *u_s_*(*t*), Equation (34), can guarantee the system output error convergence.

## 6. Experimental Results

In order to investigate the performance of the proposed controller, the following experiments were performed. The sampling frequency was chosen as 1,000 Hz. The following control parameters are chosen for the functional approximation based adaptive sliding-mode controller: the sliding surface parameter *λ* is chosen as 230 and 220 for the X axis and Y axis, respectively. The robustness parameter η can be estimated based on Equation (28). It is selected as 23,000 and 25,000 for the X axis and Y axis, respectively. In order to improve the control law chattering behavior, the sgn(*s*) function in Equation (17) is replaced by the saturation function *sat*(*s*/ф) with a boundary layer thickness *ф* = 0.1 and 0.05 for X axis and Y axis, respectively. The nominal value of the control gain *b_m_* is selected as 78.24 for X axis and 16.8 for Y axis, respectively, based on the estimation model, Equations (5) and (6). The weighting matrix *Q_f_* of the Fourier series function coefficients is set as a small constant matrix *Q_f_* = 0.01 [*I*] to increase the converging speed. The first fifteen terms of the Fourier series functions are chosen as the functional approximation basis functions.

Based on the system identification model, Equations (5) and (6), the parameters *A_m_* = 0.7202 and 0.2815, *B_m_* = 0.3588 and 0.4079, and *G_m_* = 78.24 and 16.8, are employed to design the model based sliding-mode controller for X axis and Y axis, respectively. The converging parameter, η_2_, of the sliding surface reaching condition, Equation (38), is chosen as 700 and 800 for X axis and Y axis, respectively. The parameter *λ_s_* which influences the converging slope of the sliding surface was chosen as 70 and 80 for X axis and Y axis, respectively. The values of these control parameters are not critical for practical implementation.

### 

#### Case A: The Circular Trajectory Tracking

**Figure 5.**
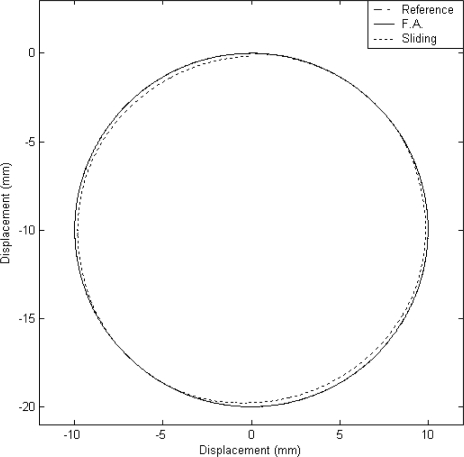
X–Y table displacement (case A).

In this case, a 2 cm diameter circular contour is designed for the two-dimensional motion control. This circular contour can be generated by the accumulation of the angles as a function of time for the X axis and Y axis. The experimental result for the tracking response of the X–Y table is shown in Figure 5.

**Figure 6.**
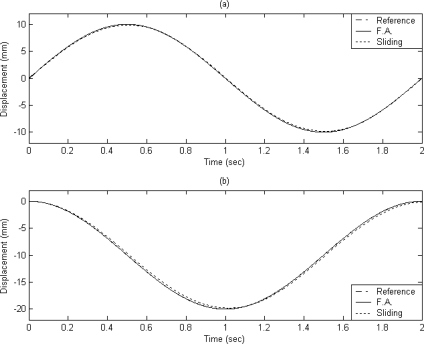
The displacements of **(a)** X axis and **(b)** Y axis (case A).

It can be observed that a good tracking response can be obtained for the X–Y table for the reference circular contour by using the proposed FAT based adaptive sliding controller. The displacements of X axis and Y axis are plotted in Figures 6(a) and 6(b), respectively.

**Figure 7.**
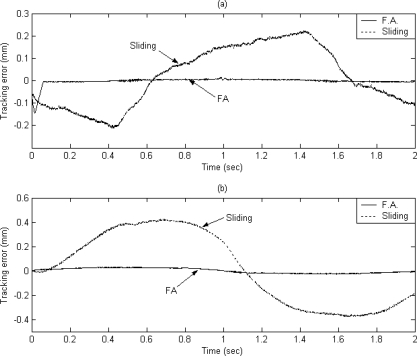
The tracking error of **(a)** X axis and **(b)** Y axis (case A).

The dashed line shows the reference circular contour signal, the solid line depicts the tracking response by using the proposed FAT based adaptive sliding controller and the dotted line denotes the tracking response by using a traditional model-based sliding-mode controller. The tracking errors of the X axis and Y axis are shown in Figures 7(a) and 7(b) for comparison. The solid line exhibits the tracking error by using the proposed FAT based adaptive sliding controller and the dotted line denotes the tracking error by using a traditional model-based sliding-mode controller. It can be observed that the maximum X axis tracking errors are 0.21 mm and 0.017 mm for the model-based sliding-mode controller and the proposed FAT based adaptive sliding controller, respectively. The maximum Y axis tracking errors are 0.42 mm and 0.028 mm for the model-based sliding-mode controller and the proposed FAT based adaptive sliding controller, respectively. The root mean square (RMS) values of the tracking error for X axis are 0.1351 mm and 0.0169 mm for the model-based sliding-mode controller and the proposed FAT based adaptive sliding controller, respectively. The RMS values of the tracking error for Y axis are 0.2991 mm and 0.0202 mm for the model-based sliding-mode controller and the proposed FAT based adaptive sliding controller, respectively.

#### Case B: The Window Contour Trajectory Tracking

**Figure 8.**
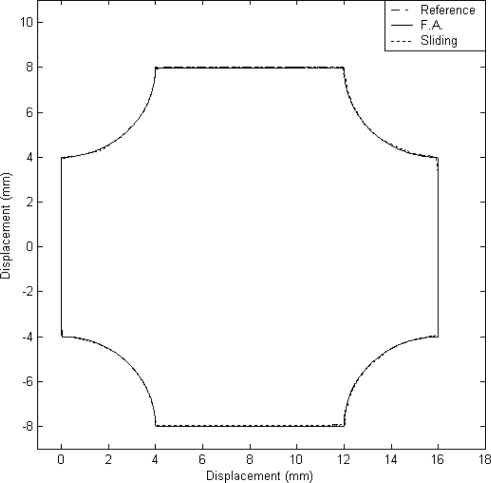
X–Y table displacement (case B).

In this case, a window contour, shown in Figure 8, was designed for the two-dimensional motion control. This window contour can be generated by the accumulation of the angles as a function of time for the X axis and Y axis. The experimental result on the tracking response of the X–Y table is shown in Figure 8. It can be observed that a good tracking response can be obtained for the reference window contour by using the proposed FAT based adaptive sliding controller.

**Figure 9.**
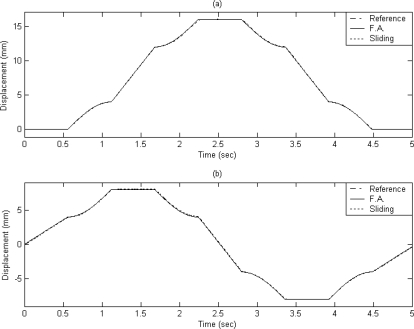
The displacements of **(a)** X axis and **(b)** Y axis (case B).

**Figure 10.**
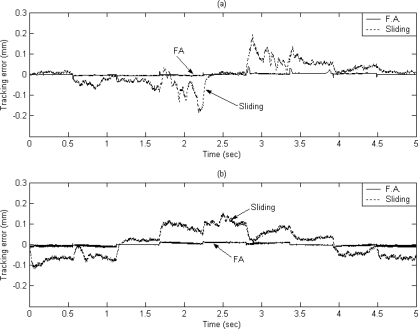
The tracking error of **(a)** X axis and **(b)** Y axis (case B).

The X axis and Y axis displacements are plotted in Figures 9(a) and 9(b), respectively. The dashed line exhibits the reference window contour signal, the solid line depicts the tracking response by using the proposed FAT based adaptive sliding controller and the dotted line denotes the tracking response by using a traditional model-based sliding-mode controller. The tracking error of X axis and Y axis are shown in Figures 10(a) and 10(b) for comparison. The solid line exhibits the tracking error by using the proposed FAT based adaptive sliding controller and the dotted line denotes the tracking error by using a traditional model-based sliding-mode controller.

It can be observed that the maximum tracking errors of the X axis are 0.2 mm and 0.013 mm for the model-based sliding-mode controller and the proposed FAT based adaptive sliding controller, respectively. The maximum tracking errors of the Y axis are 0.15 mm and 0.024 mm for the model-based sliding-mode controller and the proposed FAT based adaptive sliding controller, respectively. The root mean square (RMS) values of the tracking error for X axis are 0.0532 mm and 0.0055 mm for the model-based sliding-mode controller and the proposed FAT based adaptive sliding controller, respectively. The RMS values of the tracking error for Y axis are 0.067 mm and 0.0069 mm for the model-based sliding-mode controller and the proposed FAT based adaptive sliding controller, respectively.

## 7. Conclusions

The piezoelectric actuating system has non-linear characteristics and time-varying behavior. It is difficult to design a model-based controller for this micro-positioning system. A model-free functional approximation based adaptive sliding controller was developed and successfully employed to control a piezoelectrically actuated X–Y table system. The stability of the proposed controller is guaranteed by means of the Lyapunov theorem. The control performances of the proposed FAT based controller and a model-based sliding-mode controller were compared in this study, too. Only 15 terms of Fourier series functions are used to approximate the nonlinear time-varying function for designing a sliding-mode controller and achieving good control performance. The tracking error can be reduced to less than 0.017 mm and 0.028 mm for the X axis and Y axis with two different tracking trajectories. The tracking error is much better than that of the traditional sliding-mode controller. The proposed approach can thus be effectively applied to control a piezoelectrically actuated system.

## References

[1] Chang T, Sun X (2001). Analysis and Control of Monolithic Piezoelectric Nano-Actuator. IEEE Trans Control Syst Techn.

[2] Choi SB, Kim HK, Lim SC, Park YP (2001). Position Tracking Control of an Optical Pick-Up Device Using Piezoceramic Actuator. Mechatronics.

[3] Lin CJ, Yang SR (2006). Precise Positioning of Piezo-Actuated Stages Using Hysteresis-Observer Based Control. Mechatronics.

[4] Bashash S, Jalili N (2007). Robust Multiple Frequency Trajectory Tracking Control of Piezoelectrically Driven Micro/Nanopositioning Systems. IEEE Trans Control Syst Techn.

[5] Liaw HC, Shirinzadeh B, Smith J (2008). Robust Motion Tracking Control of Piezo-Driven Flexure-Based Four-Bar Mechanism for Micro-Nano Manipulation. Mechatronics.

[6] Song G, Zhao J, Zhou X, Abreu-García JAD (2005). Tracking Control of a Piezoceramic Actuator with Hysteresis Compensation Using Inverse Preisach Model. IEEE Trans Mechatron.

[7] Tzen JJ, Jeng SL, Chieng WH (2003). Modeling of Piezoelectric Actuator for Compensation and Controller Design. Precis Eng.

[8] Tsai MS, Chen JS (2003). Robust Tracking Control of a Piezoactuator Using a New Approximate Hysteresis Model. J Dyn Syst Meas Contr.

[9] Xue X, Tang J (2006). Robust and High Precision Control Using Piezoelectric Actuator Circuit and Integral Continuous Sliding Mode Control Design. J Sound Vib.

[10] Yeh TJ, Lu SW, Wu TY (2006). Modeling and Identification of Hysteresis in Piezoelectric Actuator. J Dyn Syst Meas Contr.

[11] Chen PC, Huang AC (2005). Adaptive Sliding Control of Non-Autonomous Active Suspension Systems with Time-Varying Loadings. J Sound Vib.

[12] Huang SJ, Chen HY (2006). Adaptive Sliding Controller with Self-Tuning Fuzzy Compensation for Vehicle Suspension Control. Mechatronics.

[13] Chen HY, Huang SJ (2008). A New Model-Free Adaptive Sliding Controller for Active Suspension System. Int J Syst Sci.

[14] Narendra KS, Annaswamy AM (1989). Stable Adaptive Systems.

[15] Utkin VI (1977). Variable Structure Systems with Sliding Modes. IEEE Trans Automat Contr.

[16] Slotine JJ, Li W (1991). Applied Nonlinear Control.

